# The Effects of Online Self-management Interventions for Patients With Mood Disorders: Protocol for a Systematic Review and Meta-analysis

**DOI:** 10.2196/45528

**Published:** 2023-03-08

**Authors:** Junggeun Ahn, Jiu Kim

**Affiliations:** 1 College of Nursing Seoul National University Seoul Republic of Korea

**Keywords:** self-management, mood disorders, online interventions, systematic review, intervention program, cognitive behavioral therapy, psychoeducation, electronic database, development, mental health

## Abstract

**Background:**

Self-management has become important as a complementary approach to the recovery of patients with mood disorders, and the need for a remote intervention program has been revealed in relation to the COVID-19 pandemic.

**Objective:**

The aim of this review is to systematically review the studies for evidence on the effects of online self-management interventions based on cognitive behavioral therapy or psychoeducation for patients with mood disorders and to verify the statistical significance of the effectiveness of the interventions.

**Methods:**

A comprehensive literature search will be conducted using a search strategy in nine electronic bibliographic databases and will include all randomized controlled trial studies conducted up through December 2021. In addition, unpublished dissertations will be reviewed to minimize publication bias and to include a wider range of research. All steps in selecting the final studies to be included in the review will be performed independently by two researchers, and any discrepancies will be resolved through discussion.

**Results:**

Institutional review board approval was not required because this study was not conducted on people. Systematic literature searches, data extraction, narrative synthesis, meta-analysis, and final writing of the systematic review and meta-analysis are expected to be completed by 2023.

**Conclusions:**

This systematic review will provide a rationale for the development of web-based or online self-management interventions for the recovery of patients with mood disorders and will be used as a clinically meaningful reference in terms of mental health management.

**International Registered Report Identifier (IRRID):**

DERR1-10.2196/45528

## Introduction

Mood disorders are emotional disturbances that can be divided into depressive disorders and bipolar disorders [1]. Depressive disorder is a disease experienced by more than 300 million people worldwide, with 13.4% of women and 8.3% of men with the disability according to the World Health Organization [2]. Bipolar disorder, which is classified as a mood disorder along with depressive disorder, affects about 2% of the world’s population and is the 10th most common disease in young adults [3,4]. For all ages, depressive disorder is the third most common disease that leads to living with a disability, and the prevalence of major depressive disorder has been significantly increasing over the years [5]. The prevalence of bipolar disorder is also rapidly increasing year after year, and in general, bipolar disorder takes an average of 5 to 10 years from the time the disease occurs to an accurate diagnosis [6,7]. Despite effective treatment, 40% of those diagnosed with bipolar disorder experience frequent recurrence, with 20%-40% of those diagnosed with major depressive disorder experiencing recurrence within one year of recovery. This suggests that mood disorders are likely to be chronic [8].

Self-management is an individual’s ability to manage the symptoms, treatment, physical and psychosocial consequences, and lifestyle changes inherent in living with a chronic condition. Promoting self-management prevents recurrence based on an understanding of one’s disease, and it is becoming important as a complementary approach to optimizing recovery from mood disorders and improving quality of life [9,10]. Self-management of mental illness can be used as a primary low-intensity intervention, especially in individuals with mild to moderate symptoms, supplementing usual clinical therapies such as drugs and psychotherapy [11]. Among the psychosocial treatments corresponding to low-intensity interventions, cognitive behavioral therapy (CBT) or psychoeducation is particularly effective in improving the psychosocial functioning, treatment adherence, and clinical course of patients with depression [12]. CBT, one of the most studied types of depression prevention intervention, is considered an effective treatment type for adults with treatment-resistant depression by reducing depressive symptoms [13,14].

The demand for and necessity of online-based interventions are increasing due to the recent influence of the COVID-19 pandemic. Recently, as the cost for health care clients increases and mental illness patients face resource limitations due to delayed treatment time, several studies have shown that computer-based or internet-based treatments are as effective as face-to-face psychotherapy in treating mood disorders [15-19]. Online-based treatment is more cost-effective than face-to-face treatment in many aspects, and it has the advantage of facilitating learning and retention because the patient has easy access to the program. In addition, the patient’s treatment progress and results can be monitored immediately, enabling prompt intervention before a crisis occurs [19].

Although the need for self-management for patients with mood disorders and the importance of online-based intervention is increasing, there is a lack of articles that have conducted systematic reviews of the literature on this topic. Therefore, to establish a theoretical basis, we will integrate the research results of online interventions based on either CBT or psychoeducation for mood disorders in a comprehensive perspective through a systematic review of the literature. Furthermore, we will verify the effect of the intervention through a meta-analysis.

## Methods

This protocol is reported in line with the PRISMA-P (Preferred Reporting Items for Systematic Review and Meta-Analysis Protocols) statements [20], and the systematic review will be conducted and reported in accordance with the PRISMA (Preferred Reporting Items for Systematic Reviews and Meta-Analyses) statement [21]. The systematic review protocol was registered under CRD42022299474 in PROSPERO.

### Identification of the Search Question

What is the evidence that online-based interventions to enhance self-management for people with mood disorders improve clinical and health outcomes?

### Search Strategy

The search strategy will be conducted by the researchers using an electronic database and search engine. A comprehensive literature search written in English will be conducted in five bibliographic databases, including PubMed, Embase, PsycINFO, CINAHL, and CENTRAL. A comprehensive literature search written in Korean will be conducted in the Research Information Sharing Service (RISS) and KoreaMED. The search will be limited to peer-reviewed journal articles available in English and Korean published until December 2021. In this study, gray literature will also be included in the review to minimize publication bias. The gray literature will be searched using Google Scholar, ProQuest Dissertations & Theses Global, and RISS.

In developing an initial search strategy based on PubMed, we adopted not only MeSH (Medical Subject Headings) terms but also synonyms and analogues as free-text terms and combined them with appropriate Boolean operators. The search strategy will be reviewed by a librarian with extensive experience in searching databases. Search strategies developed based on PubMed will be syntactically adjusted when applied to other databases. The search string will be searched using various terms such as Textbox 1. All search results will be imported from EndNote (Clarivate), a citation management software, and will be managed by researchers.

**Search strategy (PubMed).**

1. “bipolar disorder”[MeSH Terms] OR “mania”[MeSH Terms] OR (“depressive disorder”[MeSH Terms] OR “depression”[MeSH Terms]) OR “depressive disorder”[MeSH Terms] OR “mood disorders”[MeSH Terms] OR “cyclothymic disorder”[MeSH Terms] OR “bipolar*” OR “manic*”[Title/Abstract] OR “mania*” OR “hypomani*” OR “depressi*” OR “melancholia*” OR “involutional psychos*” OR (“involutional”[All Fields] AND “paraphrenia*”) OR “dysthymi*” OR “mood disorder*” OR “affective disorder*” OR “cyclothymi*”
2. “internet based intervention”[MeSH Terms] OR “internet”[MeSH Terms] OR “therapy, computer assisted”[MeSH Terms] OR “computer assisted instruction”[MeSH Terms] OR “computers, handheld”[MeSH Terms] OR “cell phone”[MeSH Terms] OR “mobile applications”[MeSH Terms] OR “smartphone”[MeSH Terms] OR “telemedicine”[MeSH Terms] OR “web browser”[MeSH Terms] OR “online systems”[MeSH Terms] OR “electronic mail”[MeSH Terms] OR “digital technology”[MeSH Terms] OR “internet*” OR “web” OR “computer*” OR “online*” OR “on line*” OR “mobile*” OR “tele monitor*” OR “telemonitor*” OR “e health*” OR “ehealth*” OR “tele health*” OR “telehealth*” OR “m health*” OR “mhealth*” OR “wireless*” OR “tablet*” OR “blackberr*” OR “android*” OR “ipad*” OR “podcast*” OR “ipod*” OR “iphone*” OR “cellphone*” OR “smart phone*” OR “smartphone*” OR “cell phone*” OR “cellular tele*” OR “app” OR “personal digital assist*” OR “pocket pc*” OR “cyber*” OR “e learn*” OR “elearn*” OR “network*” OR “electronic mail*” OR “email*” OR “e mail*” OR “digital technolog*” OR “digital electronic*”
3. “self management”[MeSH Terms] OR “self care”[MeSH Terms] OR “self manag*” OR “self care*” OR “self help*” OR “self chang*” OR “self train*” OR “self guid*” OR “self unguid*” OR “self therap*” OR “self support*” OR “self direct*” OR “self monitor*” OR “self instruct*” OR “self promot*” OR “self educat*” OR “self program*” OR “self teach*” OR “self learn*” OR “self assist*” OR “self health*” OR “self participa*” OR “self treat*” OR “self admin*” OR “self intervention*”
4. “randomized controlled trial”[Publication Type] OR “random allocation”[MeSH Terms] OR “random*” OR “randomized controlled trial*” OR “random assignment*” OR “random allocation*” OR “RCT”
5. 1 AND 2 AND 3 AND 4

### Eligibility Criteria

Studies will be included if they were conducted on adults older than 18 years, assessed those who have been diagnosed with depressive disorder or bipolar and related disorders, or who meet the diagnostic criteria. Studies will also be included if they performed online-based self-management interventions based on CBT or psychoeducation, provided sufficient information to calculate the effect size, and were conducted as a randomized controlled trial. Studies will be excluded if they were preliminary studies with no control/comparison group, performed online-based self-management interventions as part of another intervention or in combination with another intervention, or do not give access to the full text. Specifically, studies will be selected according to PICOS (Participants, Interventions, Comparisons, Outcomes, and Study Design), which is presented below.

#### Participants

The participants in the systematic review will be adults 18 years or older who have been diagnosed with or meet the diagnostic criteria for depressive disorders or bipolar and related disorders. Specifically, depressive disorders include major depressive disorder, persistent depressive disorder, and disruptive mood dysregulation. Bipolar and related disorders include bipolar I disorder, bipolar II disorder, and cyclothymic disorder.

#### Interventions

In the systematic review, we will include interventions provided in online or mobile form based on CBT or psychoeducation. Interventions should be aimed at promoting self-management in patients with mood disorders. Studies in which interventions were conducted in conjunction with or as part of other interventions will be excluded because the effectiveness of the intervention cannot be independently evaluated.

#### Comparisons

The comparison groups will be adults 18 years or older with a diagnosis of depressive disorder or bipolar and related disorders who received the following interventions: wait-list control, treatment as usual, face-to-face self-management interventions, and online self-management interventions that are not based on CBT and psychoeducation.

#### Outcomes

The primary outcomes of interest in this review will be treatment efficacy demonstrated through changes in scores for manic or depressive symptoms and quality of life. The secondary outcomes of interest will be the improvement of social functioning confirmed through the degree of change in social relationship and adaptation scores, as well as the feasibility and acceptability of the intervention calculated by the dropout rates.

#### Study Design

The study design to be included in the systematic review will be a randomized controlled trial with access to the full text. In addition, unpublished dissertations will be included in the review to minimize the risk of publication bias.

### Study Selection

All steps will be carried out independently by two authors with strict application of specific qualification criteria. Studies will be assessed by having their eligibility considered by the first author and then revised by the second author. Disagreement will be resolved through discussion. Studies retrieved from the databases listed above using a search strategy will be stored in EndNote, and then, duplicate studies will be removed through manual review by the authors and an automatic function of EndNote. A primary screening will be conducted by two authors independently, where the studies with titles and abstracts indicating that they do not meet the eligibility criteria will be removed. If at least one author identifies that a study is likely to meet the eligibility criteria, the study will not be excluded from the full-text screening. A full-text screening to select studies for final inclusion in this review will follow, which will be conducted independently by the authors. If there is disagreement at this step, the study will be reviewed and discussed together to decide whether to include it. Studies excluded from the full-text screening will be listed with the reason for exclusion, and all screening and selection processes will be reported through the PRISMA flow diagram [21].

### Data Extraction

Data will be independently extracted to electronic data extraction sheets by two authors, and they will be mutually re-evaluated. Information extracted from the studies will include trial characteristics, including first author, publication year, and participant number; intervention and control/comparison group characteristics, including diagnosis, diagnostic criteria characteristics, means of providing intervention, duration of intervention, and intervention contents; and outcome characteristics (outcome measures and results). If additional confirmation of unreported data or details is required, the study authors will be contacted to request materials. The extracted data will be saved and managed in the Excel (Microsoft Corporation) sheet (Multimedia Appendix 1). Based on the data in the Excel sheet, the diagnosis and inclusion criteria of patients to whom the intervention was applied, the type of intervention, the type of outcome measurements to evaluate the effect, and the results will be summarized and presented in a table.

### Risk of Bias and Quality Assessment

For the final selected randomized controlled trial studies, two authors will conduct an independent quality assessment using the Cochrane Risk of Bias tool [22]. This tool consists of five areas where bias can be applied to research results. The five areas are as follows: (1) risk of bias arising from the randomization process, (2) risk of bias due to deviations from the intended interventions, (3) risk of bias due to missing outcome data, (4) risk of bias in the measurement of the outcome, and (5) risk of bias in the selection of the reported result. Disagreements between the authors will be resolved through discussion. Even if the result of the quality assessment for the final selected study has a high risk of bias, the study will not be excluded.

## Results

Systematic literature searches, data extraction, narrative synthesis, meta-analysis, and final writing of the systematic review and meta-analysis are expected to be completed by 2023.

### Narrative Synthesis

We will discuss important features of the final selected study, such as publication year, number of participants, gender, age range, diagnosis and diagnostic criteria characteristics, and the type and nature of interventions provided.

### Meta-analysis

We will calculate risk ratios for dichotomous outcomes and standard mean differences (SMDs) for continuous data using complex meta-analysis software such as Review Manager (RevMan) or R (R Foundation for Statistical Computing). We will also define outcomes based on all follow-ups and adopt the most well-validated psychometric measure if there are multiple measures of the same outcome. If possible, we will contact the original author to request missing data. The statistical method of handling missing data will depend on whether the data are randomly missing. We will figure out the effect size for the intervention outcome using the SMD. The effect size will be calculated by applying either a random-effects model or a fixed-effects model according to the *I*² statistic that confirms homogeneity. In addition, we will analyze whether the direction of the effect size of each study and the confidence interval between the studies overlap through a forest plot. Funnel plots will be used to estimate the presence of publication bias. If sufficient studies are available, we will conduct the following subgroup analysis: CBT-based interventions, psychoeducation-based interventions, and CBT and psychoeducation mixed interventions.

## Discussion

This protocol has outlined detailed inclusion and exclusion criteria for determining eligible studies, which include a description of the target population and intervention of interest as well as comparisons, study design, and outcome measures. We also have mentioned our search strategy, data extraction, quality assessment, and synthesizing method such as meta-analysis to inform the direction of this study.

Depressive disorder and bipolar disorder have a high potential to become chronic diseases due to frequent relapses, so the importance of self-management is increasingly emphasized. Recently, due to the impact of COVID-19, interest in remote interventions without time and place restrictions has increased. Therefore, our research will systematically review and analyze the evidence for the effectiveness of online-based self-management interventions as a complementary approach for the recovery of patients with mood disorders. To comprehensively approach interventions for the self-management of patients with mood disorders, this study includes CBT and psychoeducation as intervention studies.

The strength of this systematic review is that it will take a standardized approach to the review method by adhering to systematic review guidelines such as the PRISMA 2020 statements and checklist. In addition, the inclusion of unpublished dissertations without limitation by year of publication will reduce publication bias and increase the range of our search. However, this review will have potential limitations in language bias, since it will only include studies published in English and Korean that the researchers can read and interpret.

Nevertheless, considering the increasing prevalence and severity of mood disorders, the effectiveness of online self-management interventions derived from this systematic review and meta-analysis can be used as clinically meaningful evidence in terms of mental health. Additionally, we believe that this study will provide the necessary data to form the theoretical basis for the development of online-based self-management interventions for the recovery of patients with mood disorders in the future.

## Appendix

Multimedia Appendix 1 Data extraction sheet.

## Abbreviations

CBT: cognitive behavioral therapy
MeSH: Medical Subject Headings
PICOS: Participants, Interventions, Comparisons, Outcomes, and Study Design
PRISMA: Preferred Reporting Items for Systematic Reviews and Meta-Analyses
PRISMA-P: Preferred Reporting Items for Systematic Review and Meta-Analysis Protocols
RISS: Research Information Sharing Service
SMD: standard mean difference

## Data Availability

Data sharing was not applicable to this paper as no data sets were generated or analyzed during this study.
