# Supplementary figures and images for: The Effects of Online Self-management Interventions for Patients With Mood Disorders: Protocol for a Systematic Review and Meta-analysis
Source: JMIR Res Protoc. 2023 Mar 8;12:e45528. doi: 10.2196/45528 (PMC10034611; doi:10.2196/45528)

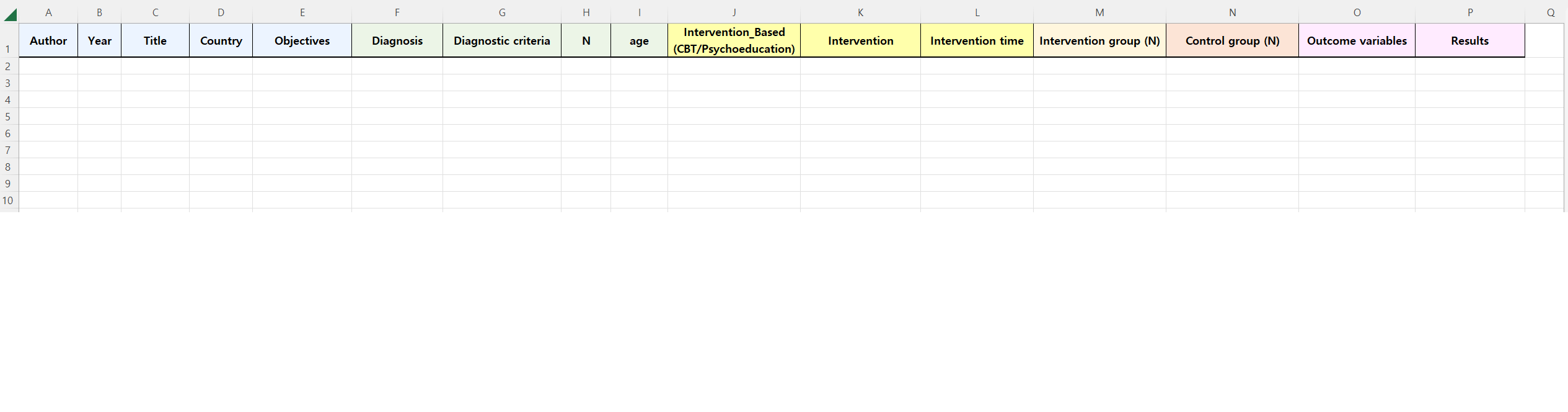

Supplement: Multimedia Appendix 1 [file resprot_v12i1e45528_app1.png]
